# Monoketone analogs of curcumin, a new class of Fanconi anemia pathway inhibitors

**DOI:** 10.1186/1476-4598-8-133

**Published:** 2009-12-31

**Authors:** Igor Landais, Sanne Hiddingh, Matthew McCarroll, Chao Yang, Aiming Sun, Mitchell S Turker, James P Snyder, Maureen E Hoatlin

**Affiliations:** 1 Department of Biochemistry and Molecular Biology, Oregon Health and Science University, Portland, USA; 2 Chemical Biology Discovery Center, Emory University, Atlanta, USA; 3 Center for Research on Occupational and Environmental Toxicology, Oregon Health and Science University, Portland, USA

## Abstract

**Background:**

The Fanconi anemia (FA) pathway is a multigene DNA damage response network implicated in the repair of DNA lesions that arise during replication or after exogenous DNA damage. The FA pathway displays synthetic lethal relationship with certain DNA repair genes such as *ATM *(Ataxia Telangectasia Mutated) that are frequently mutated in tumors. Thus, inhibition of FANCD2 monoubiquitylation (FANCD2-Ub), a key step in the FA pathway, might target tumor cells defective in ATM through synthetic lethal interaction. Curcumin was previously identified as a weak inhibitor of FANCD2-Ub. The aim of this study is to identify derivatives of curcumin with better activity and specificity.

**Results:**

Using a replication-free assay in *Xenopus *extracts, we screened monoketone analogs of curcumin for inhibition of FANCD2-Ub and identified analog EF24 as a strong inhibitor. Mechanistic studies suggest that EF24 targets the FA pathway through inhibition of the NF-kB pathway kinase IKK. In HeLa cells, nanomolar concentrations of EF24 inhibited hydroxyurea (HU)-induced FANCD2-Ub and foci in a cell-cycle independent manner. Survival assays revealed that EF24 specifically sensitizes FA-competent cells to the DNA crosslinking agent mitomycin C (MMC). In addition, in contrast with curcumin, ATM-deficient cells are twofold more sensitive to EF24 than matched wild-type cells, consistent with a synthetic lethal effect between FA pathway inhibition and ATM deficiency. An independent screen identified 4H-TTD, a compound structurally related to EF24 that displays similar activity in egg extracts and in cells.

**Conclusions:**

These results suggest that monoketone analogs of curcumin are potent inhibitors of the FA pathway and constitute a promising new class of targeted anticancer compounds.

## Background

Fanconi anemia (FA) is a multigene genetic disease characterized by developmental defects, early bone marrow failure and genomic instability leading to a high incidence of cancers [1]. At the molecular level, the FA pathway is a highly integrated DNA damage response network of proteins implicated in the repair of various DNA lesions and particularly DNA interstrand crosslinks [2,3]. The pathway is composed of a core complex of at least 10 proteins (including FANCA, B, C, E, F, G, L, M, FAAP24 and FAAP100) that function as an E3 ubiquitin ligase for the monoubiquitylation and activation of FANCD2 and FANCI [3]. Downstream proteins such as FANCD1/BRCA2, FANCJ/BRIP1 and FANCN/PALB2 have been linked to elevated risk of breast and ovarian cancers [4]. However, although the FA pathway is well-defined biochemically, its precise roles in the DNA damage response remain obscure.

The FA pathway is a potential target in anticancer therapy either through chemosensitization of tumor cells to DNA crosslinking agents such as melphalan and cisplatin [5,6] or by exploiting synthetic lethal interactions. Two genes have a synthetic lethal relationship if mutants for either gene are viable but the double mutation is lethal [7]. Targeting this particular type of genetic interaction in tumors is currently the subject of intense development due to the promising results of clinical trials using PARP inhibitors in BRCA1/2-deficient breast tumors [8,9]. High-throughput screens to identify genes displaying synthetic lethal interaction with genes frequently impaired in tumors are demonstrating the potential for discovering functional dependencies created by oncogenic mutations that may enable therapeutic intervention for cancers with "undruggable" genetic alterations such as RAS [10,11]. With regard to FA, D'Andrea and coworkers identified a set of DNA damage response genes required for the survival of FA-deficient cells including *ATM *(Ataxia Telangectasia Mutated)[12]. ATM is a major kinase involved in the sensing and repair of DNA double-strand breaks by homologous recombination [13]. Germline mutations in this gene cause the Ataxia Telangectasia cancer susceptibility syndrome [14], and *ATM *deficiencies (mutations or lack of expression) are also frequent in sporadic hematological malignancies such as chronic lymphocytic leukemia [15] and mantle cell lymphoma [16]. Because deficiency in the FA pathway is not lethal [2], specific inhibitors are expected to display low toxicity toward normal cells but kill tumor cells deficient in ATM or other genes with synthetic lethal relationships to the FA pathway.

A cell-based screen for inhibitors of FANCD2 monoubiquitylation (FANCD2-Ub) recently identified curcumin [5], a phytochemical with anticancer properties that have been linked to a variety of mechanisms including apoptosis through the NFκB pathway [17]. Efforts to develop curcumin analogs with improved solubility, stability and activity have led to the generation of a series of monoketone derivatives including EF24, a strong candidate for further drug development in cancer therapy [18-22]. We evaluated these curcumin analogs in a cell-free assay that uses *Xenopus *egg extracts to uncouple FANCD2-Ub from ongoing replication [6,23-26]. The most active compounds were subsequently tested in mammalian cells for FA pathway inhibition and synthetic lethal interactions.

## Results

### Inhibition of xFANCD2-Ub by monoketone analogs of curcumin in *Xenopus *extracts

**Figure 1:**
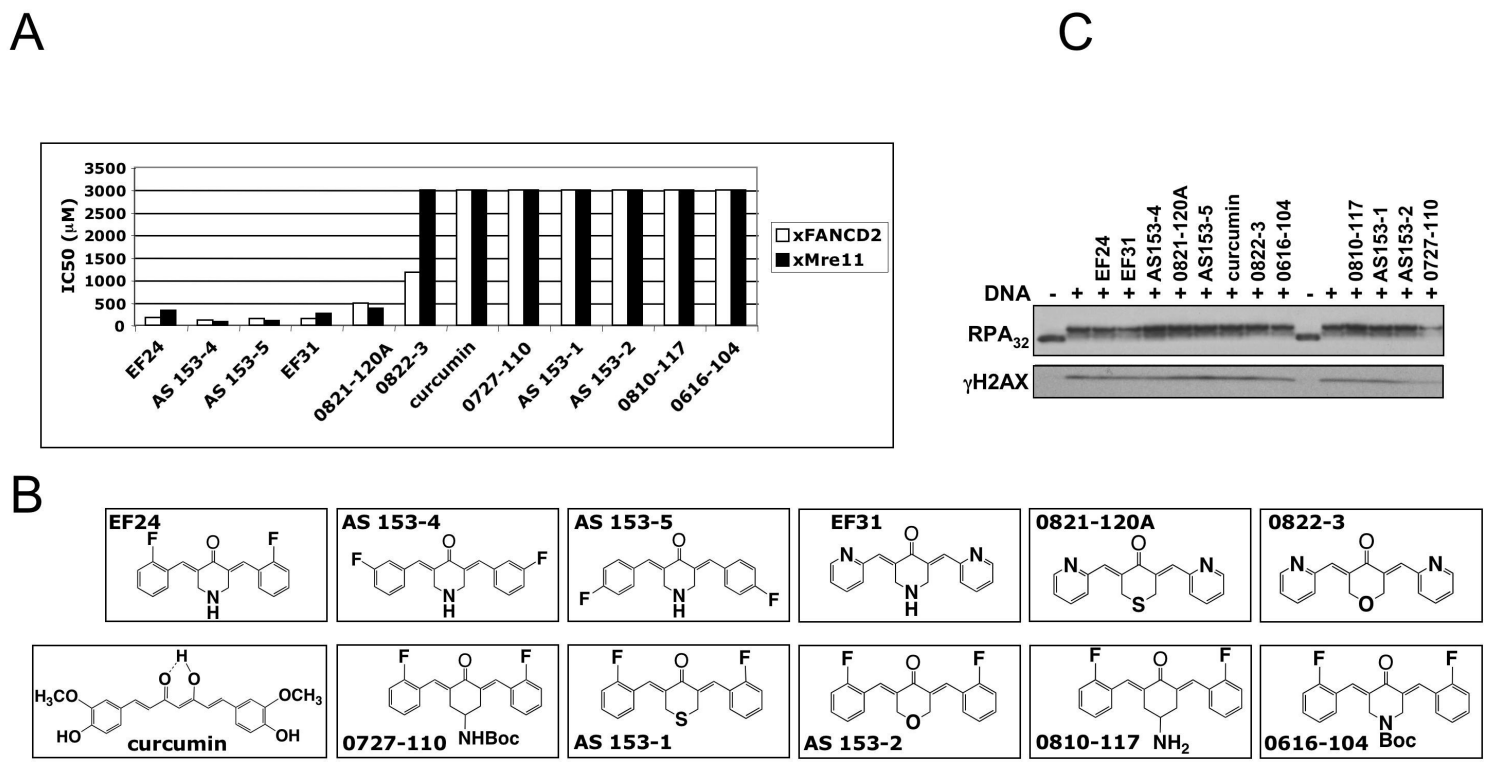
**Curcumin analogs efficiently inhibit xFANCD2 monoubiquitylation in *Xenopus *extracts**. A) Inhibition of xFANCD2-Ub and xMRE11 phosphorylation by curcumin anlogs. IC_50 _values were determined using immunoblots of plasmid-activated extracts treated with a range of concentrations for each compound (0, 10, 25, 60, 150, 400, 1000, 3000 μM). A representative experiment is shown (out of 3 repeats). B) Structure of curcumin and monoketone analogs. C) Curcumin analogs do not affect RPA_32 _and H2AX phosphorylation. Activated extracts were treated with 1 mM each compound and phosphorylation of RPA_32 _and H2AX monitored by immunoblot.

A series of monoketone analogs of curcumin [18] was evaluated in *Xenopus *egg extracts where DNA substrate-induced xFANCD2-Ub is used as a readout of FA pathway performance [6,23,24]. Phosphorylation of MRE11 (MRE11-P), a member of the MRN DNA damage repair pathway [27,28] was monitored to assess the cross-specificity of the compounds. For each compound, IC_50 _values were determined from serial dilution experiments and densitometry analysis of immunoblots. Five analogs were at least 10 fold more active than curcumin both for inhibition of xFANCD2-Ub and xMRE11-P (Fig. 1A). Structure-activity relationship (SAR) analysis (Fig. 1B) suggested that a pyridine terminal aromatic ring and a nitrogen substitution in the central ring (analogs EF24, EF31, AS153-4, AS153-5) were important for activity. Accessibility of the lone electron pair on the nitrogen atom within the ring is crucial since movement of the basic nitrogen outside the ring (0810-117) or masking by a butoxycarbonyl (Boc) group (0616-104) decreases compound activity to non-detectable levels in *Xenopus *extracts. Replacement of the central ring nitrogen with sulfur while retaining the pyridine rings (0821-120A) drops the activity only slightly, whereas the same compound with an oxygen in the central ring was only weakly active (0822-3). None of the other combinations displayed a detectable activity in *Xenopus *extracts. Phosphorylation of other proteins involved in DNA damage response (RPA_32 _[29] and H2AX, Fig. 1C) was not inhibited by the curcumin analogs.

### Mechanism of xFANCD2-Ub inhibition by EF24

To explore potential mechanisms by which curcumin analogs inhibit the FA pathway, we evaluated the integrity of the FA core complex in *Xenopus *extracts containing EF24, one of the most active analogs. In human cells, hFANCD2-Ub is reduced when FA core complex proteins are defective or absent. However we found that the integrity of the core complex was unchanged in the presence or absence of EF24 (Additional File 1 - Fig. S1). Next, we found that EF24 did not detectably interfere with the recruitment of the core complex to DNA, a crucial step for xFANCD2 activation in *Xenopus *extracts (data not shown, [24]).

**Figure 2:**
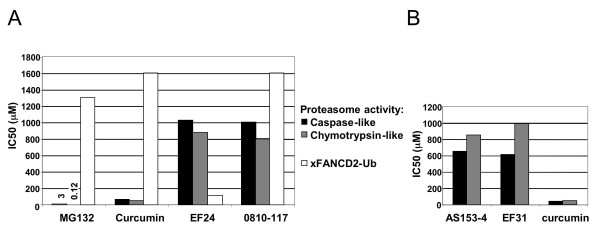
**Proteasome inhibition activity of EF24 and related curcumin analogs in *Xenopus *extracts**. A) Inhibition of caspase- and chymotrypsin-like proteasome activities and xFANCD2-Ub by MG132, curcumin and curcumin analogs. Proteasome activities and xFANCD2-Ub were monitored using fluorogenic probes and immunoblotting, respectively. IC_50 _values were determined for each compound using a range of concentrations and represented in histogram graphs. For MG132, numbers above caspase-like and chymotrypsin-like histograms indicate the actual IC_50 _values. A representative experiment is shown (out of 3 repeats). B) Inhibition of proteasome activities by EF31 and AS153-4 compared to curcumin.

The proteasome is required for the monoubiquitylation of xFANCD2 in cells [30]. As curcumin has proteasome inhibiton activities [31], we tested whether EF24 inhibits xFANCD2-Ub by this mechanism. Two proteasome activities (caspase-like and chymotrypsin-like) along with xFANCD2-Ub status were monitored in activated *Xenopus *extracts treated with various compounds (Fig. 2A). MG132 (a potent and specific proteasome inhibitor) and curcumin efficiently inhibited both proteasome activities but had only a weak activity on xFANCD2-Ub. By contrast, EF24 was 20-fold less active than curcumin for proteasome inhibition while displaying a strong activity against xFANCD2-Ub. Finally, the 0810-117 analog had weak inhibitory activities against both the proteasome and xFANCD2-Ub. To confirm the result obtained with EF24, we further tested EF31 and AS153-4, two close analogs that display a strong activity against xFANCD2-Ub (See Fig. 1A). Similar to EF24, both were 20-fold less active than curcumin for proteasome inhibition activity (Fig. 2B). Taken together, these results demonstrate that in *Xenopus *extracts EF24 and other monoketone analogs of curcumin do not inhibit the FA pathway by inhibiting the proteasome.

**Figure 3:**
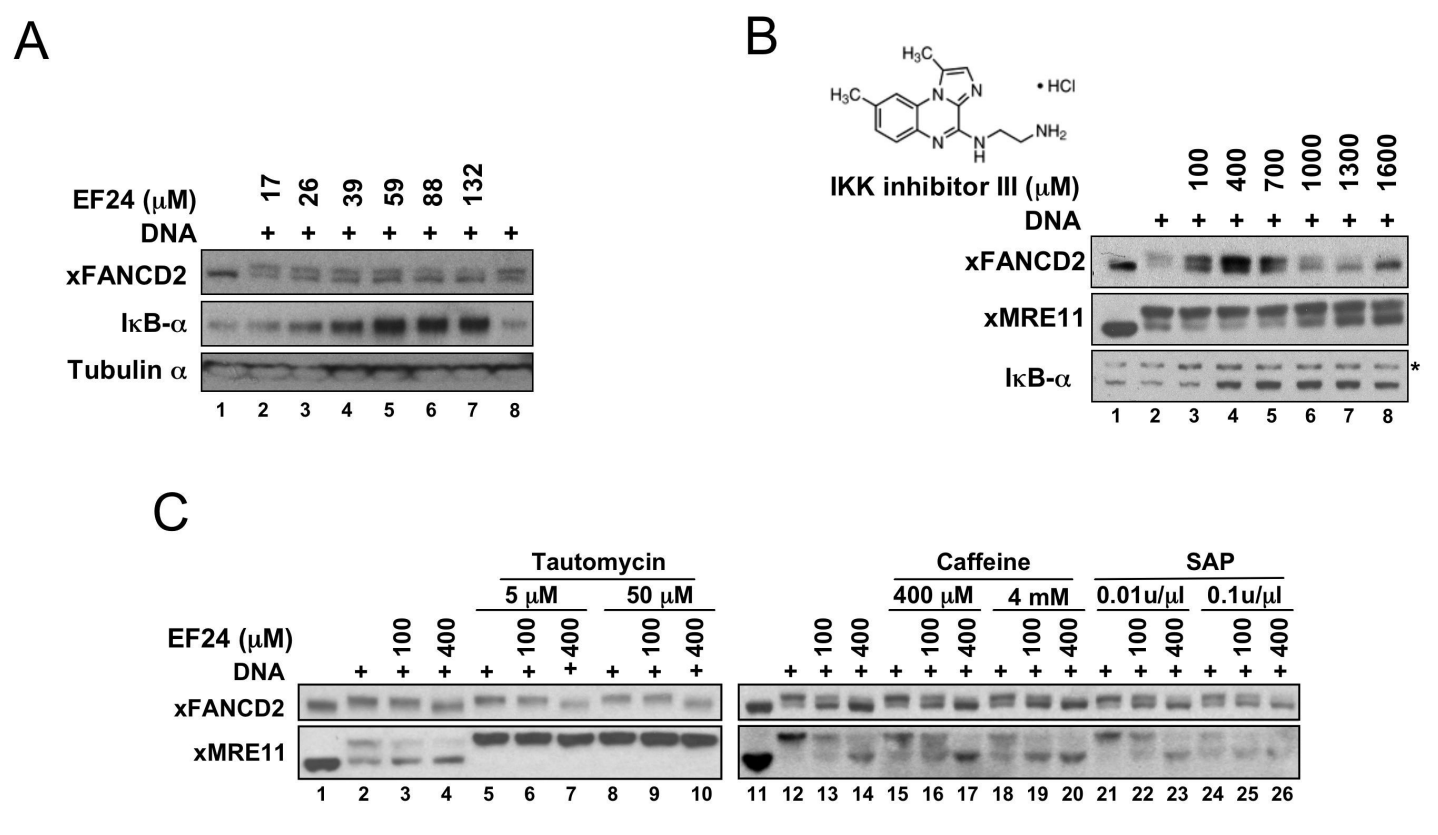
**EF24 does not inhibit xFANCD2-Ub through modulation of a phosphorylation event, but IKK inhibition might play a role**. A) EF24 inhibits the IKK kinase in *Xenopus *extracts. Extracts treated as indicated were analyzed by immunoblot using xFANCD2, IκB-α and tubulin-α antibodies. IκB-α protein level was used as readout for monitoring IKK inhibition. B) The specific IKK inhibitor compound BMS-345541 (IKK inhibitor III, Calbiochem) inhibits xFANCD2-Ub in extracts. Experiment was performed as in (A). The structure of BMS-345541 is shown. The star denotes a non-specific band used as loading control. C) EF24-dependent inhibition of xFANCD2-Ub is not affected by co-treatment with tautomycin (phosphatase inhibitor), caffeine (kinase inhibitor) and SAP (shrimp alkaline phosphatase). Extracts treated as indicated were analyzed by immunoblot using xFANCD2 and xMRE11 antibodies. xMRE11 phosphorylation status was used to monitor the efficiency of tautomycin, caffeine and SAP treatments.

IκB kinase (IKK), a critical mediator of the NFκB pathway and the cellular response to stress, has recently been identified as an important target of EF24 for its apoptosis-mediated toxicity in cancer cells [32]. Interestingly, IKK has been suggested to play a role in the activation of the FA pathway [33]. We tested whether EF24 inhibits IKK activity in *Xenopus *extracts by monitoring the accumulation of the IKK target, IκB-α (Fig. 3A). Because IκB-α phosphorylation by IKK leads to its degradation by the ubiquitin-proteasome pathway, inhibition of IKK results in the accumulation of IκB-α. For this experiment, we used extracts prepared without cycloheximide to allow for *de novo *translation of IκB-α. We found that the level of IκB-α increased upon treatment of extracts with EF24, demonstrating that EF24 inhibits IKK in *Xenopus *extracts. Next, we reasoned that if EF24 inhibits xFANCD2-Ub through inhibition of IKK, a specific IKK inhibitor should inhibit xFANCD2-Ub. After treatment of *Xenopus *extracts with increasing concentrations of BMS-345541, a specific and potent IKK inhibitor [34], xFANCD2-Ub was inhibited in a dose-dependent manner (Fig. 3B). Similar to EF24 treatment, MRE11 phosphorylation was inhibited along with xFANCD2-Ub. EF24 was 10-15 times more active than BMS-345541 for xFANCD2-Ub inhibition (EC_50_: 60 μM vs. 970 μM) and IκB-α stabilization (EC_50_: 30 μM vs. 280 μM). Interestingly, EF24 and BMS-345541 concentrations required for xFANCD2-Ub inhibition were higher than that required for IκB-α stabilization (EF24 EC_50_: 60 μM vs. 30 μM; BMS-345541 EC_50_: 970 μM vs. 280 μM, Fig. 3A and 3B), suggesting that the kinase activity of IKK is not crucial for xFANCD2-Ub in *Xenopus *extracts. To investigate this idea further, we tested whether EF24 inhibition of xFANCD2-Ub is dependent on phosphorylation (Fig. 3C). Treatment of extracts with tautomycin, a PP1/PP2A phosphatase inhibitor, resulted in the accumulation of xMRE11-P even in the presence of EF24 but had no detectable effect on xFANCD2-Ub. Treatment with caffeine, a PIKK kinase inhibitor, significantly inhibited xMRE11-P (compare lane 12 and lanes 15, 18) but co-treatment with EF24 did not alter the pattern of xFANCD2-Ub inhibition (compare lanes 12-14 and 15-17, 18-20). Since caffeine is not a general kinase inhibitor, we also used shrimp alkaline phosphatase (SAP) to dephosphorylate proteins non-specifically in extracts. Treatment with 0.1 u/μl SAP significantly reduced xMRE11-P levels (compare lanes 12 and 24) but did not affect EF24-dependent inhibition of xFANCD2-Ub (compare lanes 12-14 and 24-26). Taken together, these results suggest that EF24 might inhibit the FA pathway by targeting IKK through a mechanism that does not involve inhibition of IKK kinase activity.

### EF24 inhibits hFANCD2-Ub and hFANCD2 foci in HeLa cells

**Figure 4:**
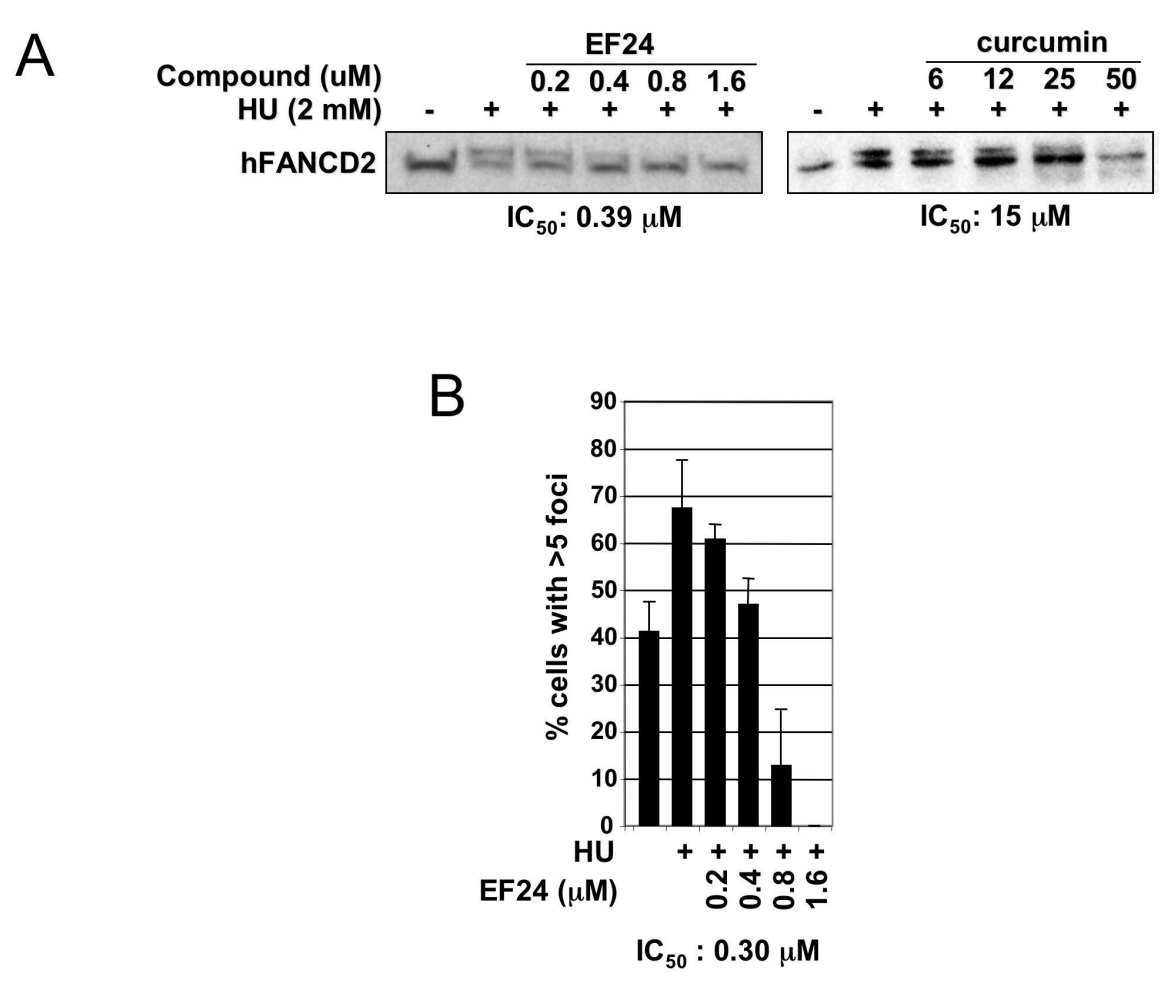
**EF24 inhibits hFANCD2-Ub and foci formation in HeLa cells and is more active than curcumin**. A) Hydroxyurea-induced FANCD2-Ub in HeLa cells is inhibited by EF24 at lower concentrations than curcumin. hFANCD2-Ub was monitored by immunoblot and densitometry analysis to determine IC_50_. B) Hydroxyurea-induced hFANCD2 foci formation is inhibited by EF24. hFANCD2 foci were detected by immunofluorescence and the percentage of cells with more than 5 foci was determined for each treatment. Histograms represent the average of 3 experiments. Error bars represent standard deviation.

We next determined whether EF24 could inhibit hFANCD2-Ub in human cells. Curcumin was tested in parallel to assess whether activity difference observed in extracts are mirrored in cells. HeLa cells were co-treated with increasing concentrations of compounds and hydroxyurea (HU), a standard method to stimulate hFANCD2-Ub (Fig. 4A, lanes 1-2, 7-8, [30]). Immunoblot and densitometry analysis revealed that EF24 is 40-fold more active than curcumin for hFANCD2-Ub inhibition (IC_50_: 0.39 μM vs. 15 μM), in line with the stronger activity of EF24 observed in extracts.

As the activity of the FA pathway is cell cycle dependent, we monitored the effect of HU and EF24 on the cell cycle by FACS analysis. We found no significant change in the cell cycle profile of cells treated with HU in combination with EF24 compared to that of untreated cells (Additional File 1 - Fig. S2), suggesting that EF24 does not inhibit hFANCD2-Ub through perturbation of the cell cycle.

To confirm inhibition of the FA pathway using another readout, we monitored the effect of EF24 on HU-induced hFANCD2 foci in HeLa cells (Fig. 4B) [29]. IC_50 _values for inhibition of foci formation were in the same range as IC_50 _values for hFANCD2-Ub inhibition, an expected result since hFANCD2-Ub but not the unmodified hFANCD2 is competent to form foci [35].

### EF24 sensitizes FA-competent cells to mitomycin C (MMC)

We tested whether EF24 is a specific inhibitor of the FA pathway by comparing its effect on a FANCA-deficient cell line (HSC72OT) and its complemented, wild-type-like counterpart (HSC72OT+A) [36] in the presence of the DNA crosslinking agent MMC. If EF24 specifically targets the FA pathway, it should sensitize FA-competent cells to MMC but not FA-deficient cells.

**Figure 5:**
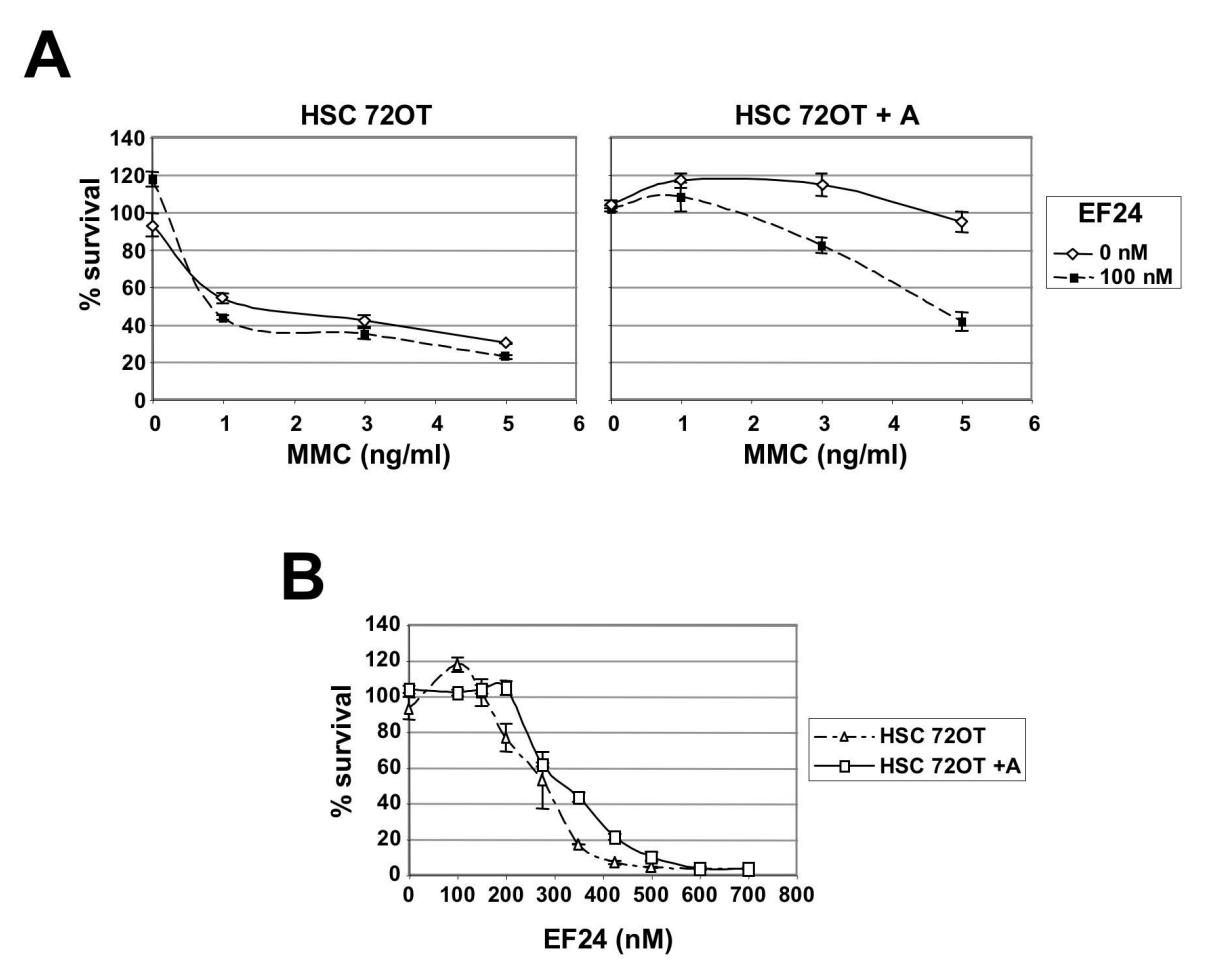
**EF24 sensitizes the HSC 72OT+A cell line to MMC but not its FA-deficient counterpart, HSC 72OT**. A) Effect of the combination of MMC and EF24 treatment on the viability of HSC 72OT and HSC 72OT+A cells. HSC 72OT (patient-derived FANCA-deficient cell line) and HSC 72OT+A (FANCA-complemented isogenic cell line) were treated with various concentrations of MMC only (solid lines) or MMC + 100 nM EF24 (dashed lines). Cell viability was measured after 3 days by MTS assay. B) Similar experiment was performed using EF24 only.

As expected, HSC72OT+A cells were more resistant to MMC compared to HSC72OT (Fig. 5A, compare solid line curves in left and right panels). 100 nM EF24, a concentration that did not affect survival in either cell line (Fig. 5B) slightly reduced the survival of MMC-treated HSC72OT cells (Fig. 5A, left panel). By contrast, the same treatment induced a significant decrease of cell survival in HSC72OT+A cells (Fig. 5A, right panel), suggesting that EF24 sensitized HSC72OT+A cells to MMC by inhibiting the FA pathway.

### Sensitivity of ATM-deficient cells to EF24 suggests a synthetic lethal effect

**Figure 6:**
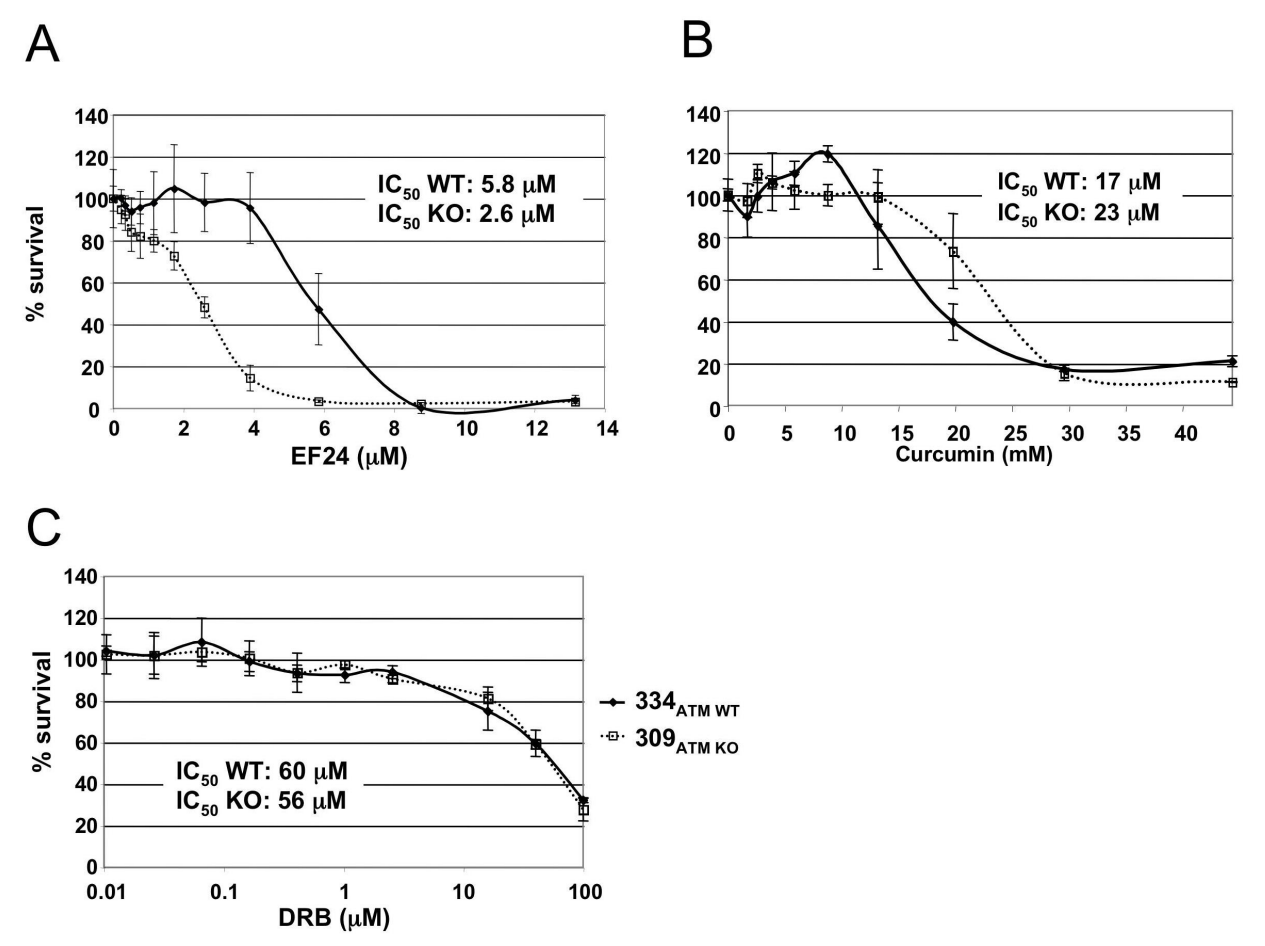
**EF24 but not curcumin or DRB is more toxic to ATM-deficient mouse kidney cells than wild-type cells**. A) 309_ATM KO _and 334_ATM WT _cells were treated with various concentrations of EF24. Cell viability was measured after 3 days by MTS assay. Each point represents the mean of 3 repeats. Error bars represent standard deviation. B) Similar experiment was performed using curcumin. C) Similar experiment was performed using DRB, a caseine kinase II inhibitor that does not inhibit the FA pathway in *Xenopus *extracts.

Kennedy et al [12] identified ATM in a screen for genes that display a synthetic lethal relationship with the FA pathway, suggesting that inhibition of the FA pathway could selectively kill ATM-deficient cells. To test this possibility, we compared the effects of curcumin and EF24 in an ATM KO mouse kidney cell line (309_ATM KO_) and its isogenic, wild type counterpart (334_ATM WT_). As expected from ATM deficiency, 309_ATM KO _cells were more sensitive to ionizing irradiation than 334_ATM WT _(Additional File 1 - Fig. S3). We next treated these cell lines with various concentrations of EF24 and measured their survival after 72 hrs. Fig. 6A shows that 309_ATM KO _cells were twofold more sensitive to EF24 than 334_ATM WT _cells (2.6 vs. 5.8 μM). In contrast, 309_ATM KO _cells were slightly more resistant than 334_ATM WT _to curcumin (23 vs. 17 μM, Fig. 6B), possibly due to the pleiotropic effects of curcumin masking the weak FA pathway inhibition activity. To rule out a general chemical sensitivity of the ATM KO cell line, we also tested the casein kinase II inhibitor DRB, a compound with no effect on the FA pathway in *Xenopus *extracts (data not shown). As expected, DRB displayed similar toxicity in both cell lines (Fig. 6C).

These results suggest that the toxicity of EF24 observed in ATM-deficient cells may be caused by a synthetic lethal effect due to the inhibition of the FA pathway in the absence of ATM.

### 4H-TTD, a novel curcumin analog with properties similar to EF24

**Figure 7:**
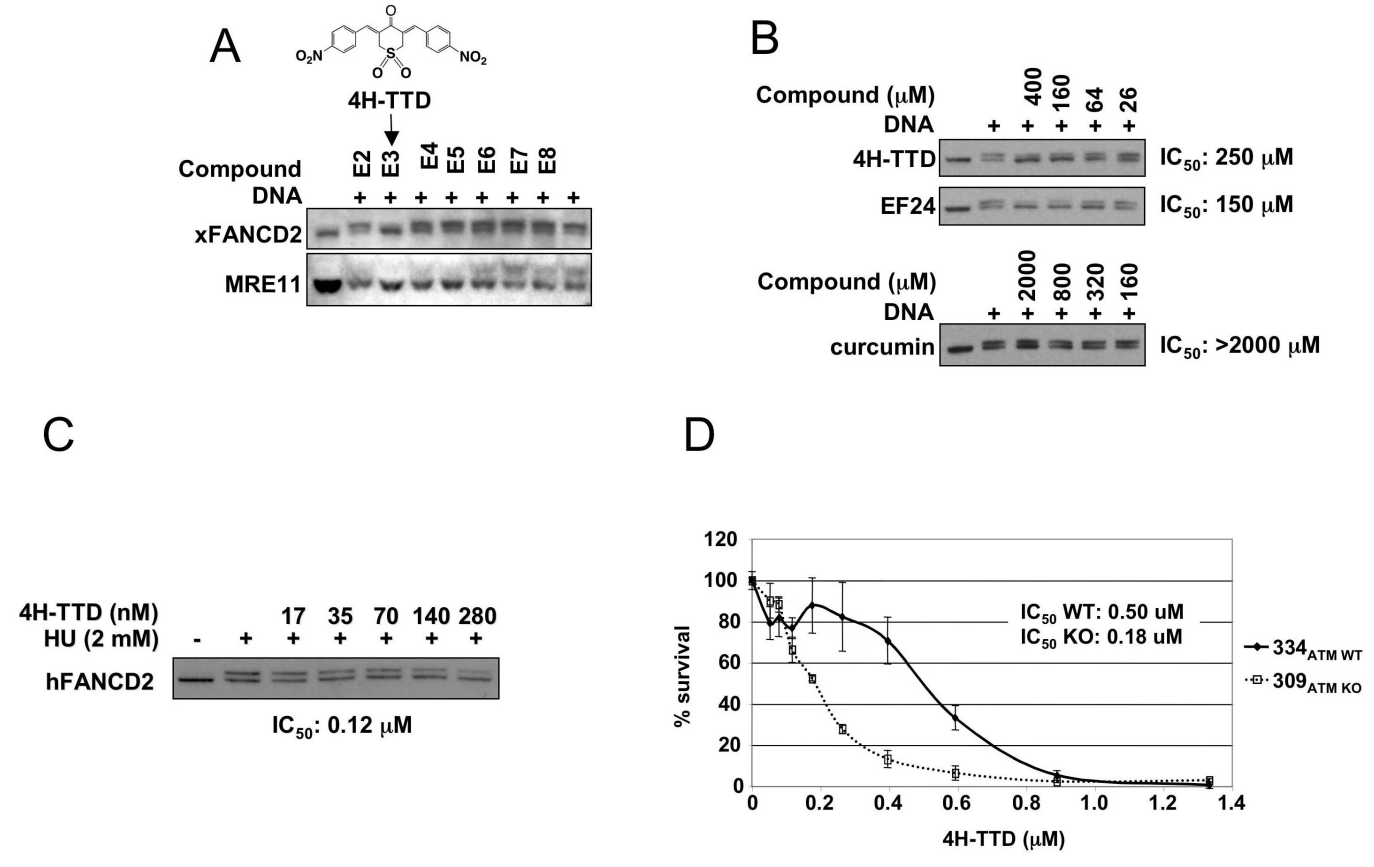
**Monoketone analogs of curcumin form a new class of FA pathway inhibitors**. A) Identification of 4H-TTD in an independent screen for FA pathway inhibitors. Each compound of row E of the NCI Challenge Set library plate was tested at 0.5 mM in *Xenopus *extracts for inhibition of xFANCD2-ub and xMRE11-P. Arrow indicates the active compound. The chemical structure of 4H-TTD is shown. B) Comparison of xFANCD2-Ub inhibition activity of 4H-TTD, EF24 and curcumin in Xenopus extracts. xFANCD2-Ub IC_50 _were determined as in Fig. 1A. C) 4H-TTD inhibits the FA pathway in HeLa cells. IC_50 _was determined as in Fig. 4A. D) 4H-TTD is more toxic to 309_ATM KO _than 334_ATM WT _cells.

We previously identified several small molecule inhibitors of the FA pathway using the *Xenopus *cell-free assay [6]. Structural analysis revealed that one of the compounds (E3 of the Challenge Set library, NCI) is a curcumin analog that resembles EF24 (Fig. 7A). The structure incorporates a sulfone moiety instead of an amine in the central ring and *p*-nitro substitution in the terminal phenyl rings instead of *o*-fluoro groups. Its chemical name, 4H-Thiopyran-4-one, tetrahydro-3,5-bis [(4-nitrophenyl)methylene]-,1,1-dioxide, was abbreviated to 4H-TTD for convenience. Comparison with curcumin and EF24 revealed that 4H-TTD is slightly less active than EF24 for FA pathway inhibition in Xenopus extracts (IC_50_: 250 μM vs. 150 μM, Fig. 7B). In HeLa cells, 4H-TTD inhibited HU-induced hFANCD2-Ub at sub-micromolar concentrations, similar to EF24 (IC_50_: 0.12 μM, Fig. 7C). We next tested 4H-TTD activity in the survival assay described in the previous paragraph (Fig. 6). Like EF24, 4H-TTD displayed more toxicity in 309_ATM KO _cells, with a 3-fold difference compared to WT cells (0.18 vs. 0.5 μM, Fig. 7D). The overall cell toxicity was in the nanomolar range, about 10 times lower than EF24, suggesting that 4H-TTD has a general cellular toxicity greater than EF24.

These results underscore the use of *Xenopus *egg extracts as a useful screening strategy to rapidly identify inhibitors of the FA pathway and further validates this class of compounds as potent FA pathway inhibitors and potential anticancer agents for synthetic lethal relationship-based targeted therapy.

## Discussion

DNA damage response pathways have recently been the target of considerable efforts in oncology research. This is due in part to the successful treatment of *BRCA*-deficient tumors with PARP1 inhibitors [8,9], which demonstrated that taking advantage of the synthetic lethal relationship between two DNA damage genes is a valid approach for the development of novel targeted therapies in oncology [7]. Two parameters are important for a synthetic lethal interaction to be exploited therapeutically against cancers: first, functional deficiency of one of the genes has to be a causative event of the tumorigenesis. Impairment of DNA damage responses, which is strongly suspected to be one of the most common initial event during tumorigenesis [37], fits this requirement. The second condition is that the target of the therapeutic agent is not an essential gene, hence limiting toxicity and increasing the therapeutic window. Many DNA damage response pathways are partially redundant, explaining why one defect is usually well tolerated at least in the short term, *e.g. PARP1 *[7,38].

Synthetic lethal interactions between the FA pathway and other DNA damage response genes such as *ATM*, *PARP1 *and *NBS1 *have been identified recently [12], stimulating interest in determining if FA pathway inhibitors would selectively target tumor cells deficient in these genes. For instance, *ATM *mutations are highly prevalent (approx. 50%) in mantle cell lymphoma patients [16], raising the possibility that these tumors might be selectively treated with a FA pathway inhibitor.

The first identified FA pathway inhibitor was curcumin [5]. To improve potency and specificity, we compared the FA pathway inhibition activity of a series of monoketone analogs of curcumin [18] in *Xenopus *extracts, and identified EF24 and 4 other compounds as potent FA pathway inhibitors. Similar to DDN and other inhibitors we previously identified [6] and data not shown), curcumin analogs displayed similar activity against MRE11 phosphorylation but did not inhibit RPA_32 _and H2AX phosphorylation. This finding will be important practically for the design of a high-throughput assay, allowing rapid discrimination between specific FA pathway inhibitors and those that also inhibit MRE11 phosphorylation.

### Structure-activity relationships

Structural similarity of EF24 and 4H-TTD confirmed that monoketone analogs of curcumin form a new class of FA pathway inhibitors. Within the same structural template, heteroatom ring replacements and a subset of terminal aromatic ring substituents appear to account for the potency of a given compound. With respect to the central six-membered ketone for example, the piperidone derivatives (EF24, EF31, AS153-4, and AS153-5) are much more potent than the corresponding thio-pyran analog (AS153-1). However, decorating the nitrogen of the piperidone with a strong electron withdrawing group that diminishes the basicity of the nitrogen, for instance the Boc group, causes complete loss of activity (0616-104). Switching from piperidone to pyran or cyclohexanone likewise decreases activity to undetectable levels in Xenopus extracts (AS153-2 and 0810-117). By contrast, the central sulfone of 4H-TTD restores activity perhaps by virtue of its unique ability to serve as a proton acceptor on both faces of the central ring. With respect to terminal ring substitution, the moieties that deliver the most potent analogs are F, OH and the pyridine nitrogen (Fig. 1) [18]. Aromatic nitro groups coupled with a central ring sulfone as represented by 4H-TTD (Fig. 7A) represents a novel combination. This emerging SAR provides a direction for further optimization. For example, it will be instructive to see if combining nitro groups with a piperidone ring or fluoro substituents with sulfone will either match or enhance the observed activities.

While exploring the mechanism of FA pathway inhibition, we found that EF24 is a weak proteasome inhibitor. The more effective proteasome inhibition by curcumin (Fig. 2) may be due to structural features absent in the monocarbonyl analogs. Three prominent structural elements that differentiate the two chemical classes are the central rigidifying ring in the analogs, the presence of the central three-carbon unit bearing two oxygens and the phenolic oxygens in curcumin. The antioxidant properties of curcumin are generally attributed to the radical scavenging abilities of the phenolic OH groups [39] and the central 1,3-diketone functionality [40]. Thus, either radical scavenging or a change in molecular architecture or both may be the basis for curcumin's superior activity against the proteasome. This result implies that EF24 has a spectrum of activities narrower than curcumin.

### Inhibition of the FA pathway in cells

With activity in the nanomolar range in cells, EF24 and 4H-TTD are the most active FA pathway inhibitor identified so far [5,6,30]. As previously noted [6], EF24 and 4H-TTD effective concentrations were 2 to 3 orders of magnitude lower in cells than in extracts, probably because of the strong quenching occurring in extracts due to high proteins and lipids content [41].

We next tested whether inhibition of the FA pathway by EF24 could be exploited to target cells using sensitization and synthetic lethal interaction approaches. Using a combination strategy [6], we found that a non-toxic dose of EF24 sensitized FA-competent cells to the DNA crosslinking agent MMC. EF24 failed to sensitize isogenic FA-deficient cells to MMC, suggesting that inhibition of the FA pathway rather than pleiotropic activity was responsible for the sensitization phenotype observed (Fig. 5). Hence EF24 might be an interesting lead as a chemosensitizer for DNA crosslinking agents such as MMC and platinium compounds.

Next, EF24 and 4H-TTD were used as single agents in congenic cell lines differing in their ATM status. We found that both compounds were significantly more toxic to ATM KO cells than WT cells. These results are consistent with a model where inhibition of the FA pathway by curcumin analogs triggered a synthetic lethal effect in ATM-deficient cells [12]. The failure of curcumin to display the same effect could be due to the fact that its weak activity against the FA pathway is masked by the activity toward numerous other targets, for instance the proteasome.

### Mechanism of FA pathway inhibition

EF24 did not inhibit the FA pathway by well-known mechanisms such as disrupting the integrity of the FA core complex [42], impairement of recruitment of FA proteins to DNA substrates [24] or proteasome inhibition [30]. Surprisingly, proteasome inhibition did not affect xFANCD2-Ub in *Xenopus *extracts, suggesting that the proteasome is not involved in the basic activation of the FA pathway. Rather, it may be required in the more complex cellular environment to overcome the chromatin barrier or to respond to specific DNA lesions [30]. Importantly, this finding further highlights the use of the Xenopus extracts-based screening assay to identify compounds that are more likely to target the core FA pathway rather than general regulators. Underscoring this notion, monoubiquitylation of FANCD2 is also independent of phosphorylation in DNA-stimulated extracts (Fig. 3C[24]).

Interestingly, Aleo E. et al [43] identified 4H-TTD (called "G5" in their work) in a screen for compounds that trigger caspase activation in a BCL-2 dependent and caspase 9-independent manner. 4H-TTD did not inhibit proteasome activity, confirming the data obtained with EF24 and related analogs. Instead, 4H-TTD exhibited ubiquitin isopeptidase/deubiquitinase (DUB) inhibition activity. Structural analysis further revealed a molecular determinant shared with known ubiquitin isopeptidase inhibitors [43] that is also present in EF24, raising the possibility that monoketone analogs of curcumin could inhibit FANCD2- Ub through a novel mechanism involving DUB inhibition independently of the proteasome.

During this study, we found that EF24 inhibits the phosphorylation of xMRE11 and xFANCM in Xenopus extracts (Fig. 1, 3 and Additional File 1 - Fig S1). Since both proteins are the target of the DNA damage signaling and checkpoint kinases ATM and ATR [28,44], we tested whether EF24 treatment affected the phosphorylation status of a classic ATR target, CHK1 [45]. Reprobing the EF24 blot shown in Fig. 7B with a phospho-CHK1-specific antibody revealed that CHK1-P was inhibited at the highest EF24 concentration tested (400 mM, Additional File 1 - Fig. S4), suggesting that this curcumin analog is a bona fide but weak ATR inhibitor. Since FANCD2 monoubiquitylation has been shown to be dependent on ATR both in mammalian cells [46,47] and in replicating Xenopus extracts [23,26,44], it was therefore tempting to hypothesize that EF24 inhibits FANCD2-Ub through ATR inhibition. However our lab has clearly demonstrated that FANCD2-Ub is not dependent on ATR in non-replicating, DNA substrate-stimulated extracts: chemical inhibition of ATR by caffeine or depletion of ATR and its essential partner ATRIP have no detectable effect on FANCD2-Ub in this setting (Fig. 3C and [24,44]). Taken together, these data suggest that EF24 might be a weak ATR inhibitor, but this activity does not account for FANCD2-Ub inhibition in non-replicating extracts.

An alternative hypothesis emerged from the fact that one of the cellular targets of curcumin and EF24 is the IκB kinase complex (IKK), a major component of the NF-κB pathway. Otsuki et al [33] showed that IKK interacts with the FA core complex, raising the possibility that EF24 might inhibit the FA pathway by targeting IKK. This hypothesis is in line with the fact that curcumin has a weaker inhibitory activity than EF24 toward both IKK and xFANCD2-Ub (Fig. 1, Fig. 4 and [32]). Interestingly, we found that the specific IKK inhibitor BMS-345541 [34] inhibited xFANCD2-Ub in a manner similar to EF24, i.e. xFANCD2-Ub inhibition occurred at higher concentration than IkB-α stabilization (Fig 3A, B). This differential effect is reminiscent of the fact that BMS-345541 is 10-fold more active on IKK-2 (IKKβ) than on IKK-1 (IKKα), maybe because allosteric binding induces a different conformational change in the two IKK subunits [34]. The latter result also suggests that the kinase activity of IKK is not required for plasmid activation of the FA pathway in extracts. The fact that general phosphorylation modulators did not interfere with EF24 (Fig. 3C) provided an indirect confirmation of this hypothesis. Additional experiments are needed to explore the potential link between EF24, IKK and the FA pathway.

Like curcumin, the mimics depicted in Figure 1 are pleiotropic agents with activities as diverse as mitochondrial redox-mediators [19], tubulin dynamics disrupters [22], kinase inhibitors [32], glioma cell radiosensitizers [48] and angiogenesis blockers [21]. Although their multiplicity of actions is complex, predictive pharmacological models [49] and optimization [50] are emerging within systems biology. The discovery that monoketone analogs of curcumin form a new class of FA pathway inhibitors not only underscores their potential for low toxicity targeted cancer therapy, it also adds to the still incomplete network of beneficial biological actions exerted by these multi-tasking agents.

## Conclusions

**Figure 8:**
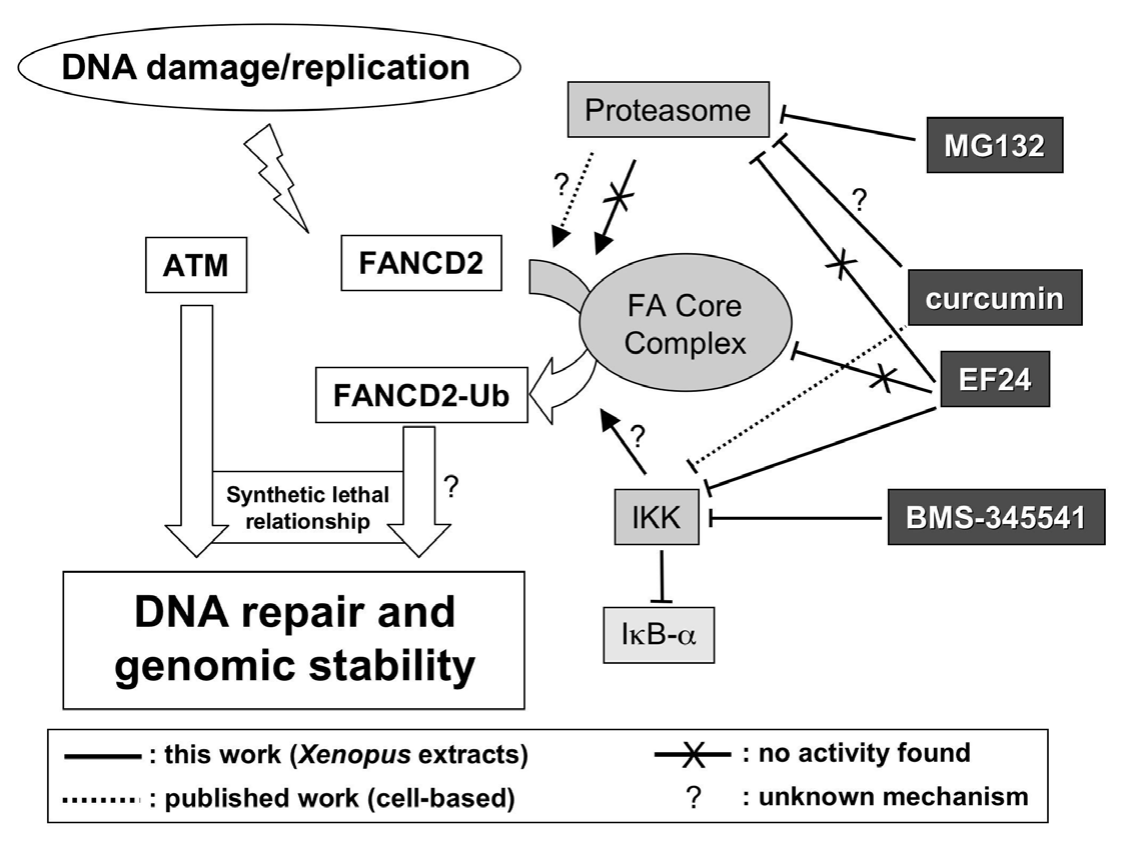
**Summary of the mechanistic findings presented in this paper**. The ATM and FA pathways (white boxes/arrows) are two DNA damage response pathways that display synthetic lethal relationship. Inhibition of the FA pathway may therefore be used as a targeted therapy to selectively kill ATM-deficient tumors. Several enzymatic complexes have been proposed to modulate the FA pathway (light grey boxes). Monitoring the effect of the compounds used in this study (dark grey boxes) toward those enzymatic activities suggests that (i) in contrast with cell-based assays, the proteasome is not required for FA pathway activation in Xenopus extracts, (ii) in contrast with curcumin, EF24 is a weak proteasome inhibitor, (iii) EF24 does not inhibit the FA pathway through disruption of the core complex, and (iv) curcumin, EF24 and BMS-345541 might inhibit the FA pathway through inhibition of IKK.

Using curcumin as a lead we identified EF24, a monoketone analog with improved activity and specificity toward the FA pathway. An independent screen identified 4H-TTD, a structurally related compound with similar activities, suggesting that monoketone analogs form a new class of FA pathway inhibitors. Fig. 8 summarizes the main mechanistic findings of the study.

To our knowledge, this is the first report suggesting that small molecule inhibition of the FA pathway may specifically target ATM-deficient cells. Additional experiments are under way to confirm this synthetic lethal effect and to test whether these compounds are effective in human ATM-deficient cancer cells as well.

## Methods

### Chemicals and antibodies

Monoketone analogs of curcumin were prepared as previously described [18]. Pure curcumin, MG132 (Calbiochem), IKK inhibitor III (BMS-345541, Calbiochem), DRB (Calbiochem), 4H-TTD (NSC 144303, NCI/DTP Open Chemical Repository, http://dtp.nci.nih.gov) were resuspended in DMSO, tautomycin (Calbiochem) in EtOH, caffeine and hydroxyurea (Sigma) in H_2_O. Shrimp alkaline phosphatase was from Fermentas. Antibodies against xFANCD2, xFANCG, xFANCA and hFANCD2 were described previously [6]. Antibodies against γH2AX and IkB-α were from Bethyl laboratories (#A300-081A) and cell Signaling (#9242), respectively. Antibodies against xRPA, xMre11 and hFANCD2 (for immunofluorescence) were kind gifts of K. Cimprich, J. Gautier and K.J. Patel, respectively.

### *Xenopus *cell-free assay and immunoblotting

Preparation of *Xenopus laevis *low-speed extracts and the FA pathway assay were described previously [6].

### Proteasome assay

Fluorogenic peptides (Proteasome Substrate Pack #PW9905) specific for the chymotrypsin-like and caspase-like activities of the proteasome were incubated in *Xenopus *extracts treated with serial dilutions of various compounds. Fluorescence emitted by proteasome cleavage of the peptides was monitored using a fluorometer (FluoStar Galaxy, BMG Labtech) with 380 nm and 460 nm excitation and emission filters, respectively. Experiments were repeated at least twice. Due to the large variability of activity between extract batches, one representative experiment is shown.

### Cell lines and cell culture

HeLa cells were grown in DMEM medium supplemented with 10% serum in humidified 5% CO2 atmosphere. ATM-deficient and ATM-proficient kidney cells from congenic adult mice (309_ATM KO _and 334_ATM WT_) [51] were grown in the same medium.

### hFANCD2 ubiquitylation assay and immunofluorescence microscopy in HeLa cells

hFANCD2 ubiquitylation assay and immunofluorescence assays in HeLa cells have been described elsewhere [6].

### Survival assays

HSC72OT and HSC72OT+A cells were seeded at 5,000 per well in 96-well plates, and treated 24 hrs later with various concentrations of compounds or DMSO (1 μl in 100 μl medium). 309_ATM KO _and 334_ATM WT _cells were seeded at 7,000 per well and treated immediately. Cell survival was measured after 3 days using the CellTiter 96 AQueous One Solution Cell Proliferation Assay (Promega) following the recommendations from the manufacturer. All experiments were done in triplicate.

### Calculation of IC_50 _values

Fitted curves were determined from experimental data using IgorPro. The concentration of compound that induces 50% inhibition of the measured endpoint (i.e. FANCD2 ubiquitylation, Mre11 phosphorylation and cell survival) was then plotted on the fitted curve.

## Competing interests

The authors declare that they have no competing interests.

## Authors' contributions

IL designed the study, performed experiments in Xenopus extracts, immunofluorescence, FACS analysis and drafted the manuscript. SH performed cell-based hFANCD2 ubiquitylation assays and cell survival assays. MMC performed cell survival assays. AS prepared curcumin analogs in Fig. 1 and helped draft the manuscript. CY prepared fresh samples of 4H-TTD. MST prepared ATM WT and KO cells and helped draft the manuscript. JPS chose the analogs for chemical testing and wrote the chemical section. MEH conceived the study, coordinated lab efforts and helped draft the manuscript. All authors read and approved the final manuscript.

## Supplementary Material

Additional file 1 **Supplementary Figures**. This file contains four supplementary figures. Figure S1: EF24 does not inhibit the FA pathway through disruption of the core complex in Xenopus extracts. Figure S2: Combination of EF24 with HU does not significantly alter the cell cycle compared to untreated cells. Figure S3: 309_ATM KO _cells are more sensitive to ionizing irradiation than 334_ATM WT _cells. Figure S4: EF24 inhibits phosphorylation of CHK1 (CHK1-P) in DNA-stimulated Xenopus extracts. Click here for file
