# Supplementary material for: Monoketone analogs of curcumin, a new class of Fanconi anemia pathway inhibitors
Source: Mol Cancer. 2009 Dec 31;8:133. doi: 10.1186/1476-4598-8-133 (PMC2807854; doi:10.1186/1476-4598-8-133)
Supplement: Additional file 1 — Supplementary Figures. This file contains four supplementary figures. Figure S1: EF24 does not inhibit the FA pathway through disruption of the core complex in Xenopus extracts. Figure S2: Combination of EF24 with HU does not significantly alter the cell cycle compared to untreated cells. Figure S3: 309ATM KO cells are more sensitive to ionizing irradiation than 334ATM WT cells. Figure S4: EF24 inhibits phosphorylation of CHK1 (CHK1-P) in DNA-stimulated Xenopus extracts. [file 1476-4598-8-133-S1.PDF]

Figure S1

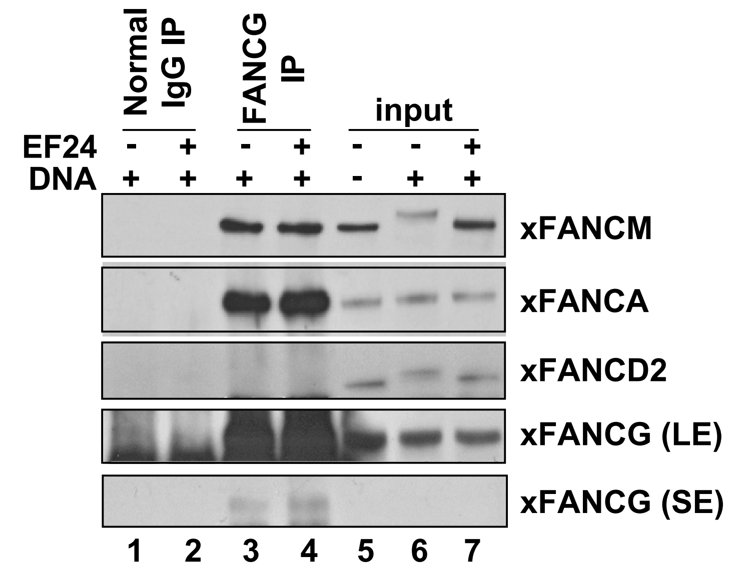

EF24 does not inhibit the FA pathway through disruption of the core complex in Xenopus extracts. Xenopus extracts were treated with 1mM EF24 and core complex integrity was monitored by co-immunoprecipitation of FANCM and FANCA with FANCG (lanes 3-4). Normal IgG was used as negative control for IP (lanes 1-2). Input corresponds to 5% of IP volume. LE, long exposure; SE short exposure. Co-immunoprecipitation of 3 members of the core complex (FANCA, FANCM and FANCG) was similar in the presence or in the absence of 1 mM EF24 (Fig. 2A lanes 3, 4) even though xFANCD2-Ub was completely inhibited by EF24 (lane 7). As expected, xFANCD2, which is not a member of the core complex, did not co-immunoprecipitate with xFANCG (lanes 3, 4).

Materials and Methods. 40 µl of egg extract (2 mg total protein content) were incubated with plasmid DNA (150 ng/µl) and 1 mM EF24 (or DMSO) for 20 min at room temperature before addition of 1 ml lysis buffer (10 mM Tris pH 7.4, 150 mM NaCl, 1% NP40, 0.5% deoxycholate, 1mM EDTA, 0.5 mg/ml Pefabloc, 1mM DTT). 10µl of rabbit polyclonal antibody against xFANCG was added and samples were mixed by rotating overnight at 4°C. 100 µl of pre-swelled and washed (50% slurry in PBS) proteinA-sepharose beads (GE Healthcare) were added and rotated for 30 minutes at 4°C. Beads were pelleted by centrifugation, washed 3 times with lysis buffer and proteins were eluted by boiling in protein loading buffer.

Figure S2

A

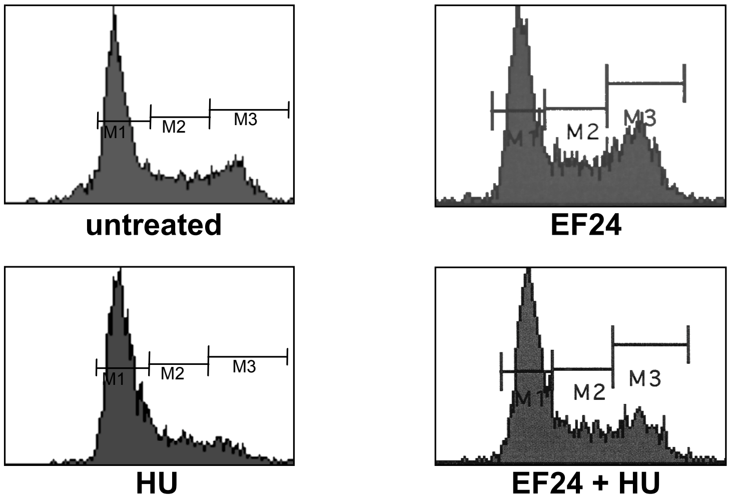

B

	G1	S	G2/M
**untreated**	59	20	21
**HU**	67	22	11
**EF24**	48	22	30
**EF24 + HU**	59	21	20

Combination of EF24 with HU does not significantly alter the cell cycle compared to untreated cells. Hela cells were treated for 8 hrs with 2mM HU and/or 2 mM EF24 as indicated, stained with PI and subjected to DNA content analysis by FACS. **(A)** DNA content profiles are shown with M1: 2N DNA (G1 phase), M2: 2N<DNA<4N (S phase), M3: 4N DNA (G2/M phases). **(B)** Percentage of cells in each phase for each treatment. A representative experiment (from 2 repeats) is shown.

As the activity of the FA pathway is cell-cycle dependent, we tested whether the effect of EF24 on HU-induced FANCD2-L correlated with differences in cell cycle profile. FACS analysis indicated that HU treatment strongly decreased the population of G2/M cells, consistent with the fact that HU efficiently blocks replication. On the other hand, cells treated with EF24 accumulated in G2/M phase. However, when these compounds were combined with HU, the profile did not significantly change compared to that of untreated cells, suggesting that EF24 does not inhibit FANCD2-L through perturbation of the cell cycle.

Materials and Methods. For FACS analysis, cells were trypsinized, fixed in 70% EtOH and stained for 16 hrs at $4^{o}C$ in propidium iodide (PI) solution (40 µg/ml PI, 0.2 mg/ml RNAse A, 0.1% triton in PBS). For each point, 10000 gated cells were counted using a FACSCalibur flow cytometer (BD Biosciences).

Figure S3

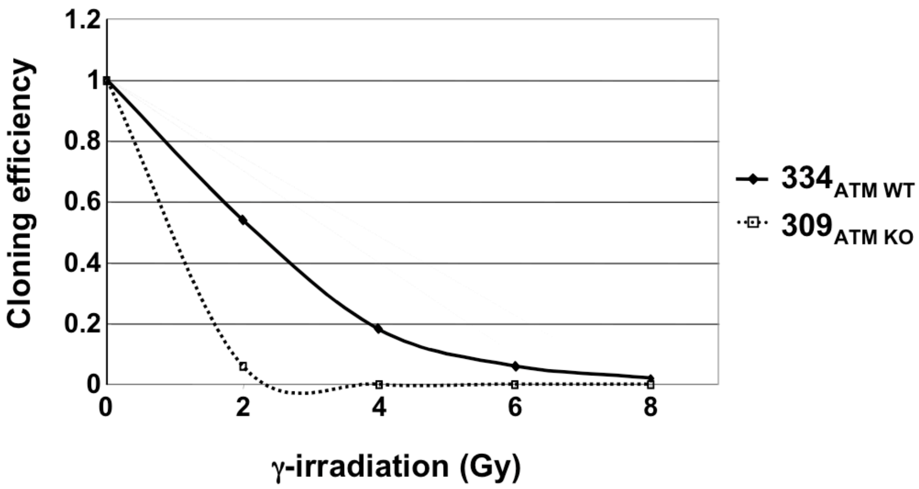

$309_{ATM\ KO}$ cells are more sensitive to ionizing irradiation than $334_{ATM\ WT}$ cells. Viability of cells γ-irradiated as indicated was assessed by clonogenic assay.

Materials and Methods. $309_{ATM\ KO}$ and $334_{ATM\ WT}$ cells were plated at a density of 500-4000 cells per 100 mm plate and irradiated with 0, 2, 4, 6 or 8 Grays (G). After 10 days, crystal violet-stained colonies containing more than 50 cells were counted. Colony formation efficiency (number of colonies/number of initial cells) of non-irradiated plates was set to 1.

Figure S4

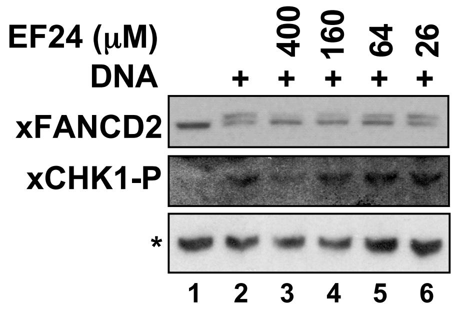

EF24 inhibits phosphorylation of CHK1 (CHK1-P) in DNA-stimulated Xenopus extracts. The EF24 blot shown in Fig. 6B was reprobed with a phospho-CHK1 specific antibody. Inhibition of plasmid-induced xCHK1-P was apparent at 400 μM EF24 treatment. (*) A non-specific band was used as loading control. See legend of Fig. 7B for experimental details.
